# Short-Term Fascial Circulation Exercise Modulates Task-Related Prefrontal Oxygenation During Executive Tasks in Older Women: An fNIRS Pilot Study

**DOI:** 10.3390/life16030458

**Published:** 2026-03-11

**Authors:** Suyoung Hwang, Yae-Hyun Leem, Moon Hee Kim, Eun-Surk Yi

**Affiliations:** 1 Department of Exercise Rehabilitation & Welfare, Gachon University, Incheon 21936, Republic of Korea; harriett0059@gmail.com (S.H.); yaehyun@hanmail.net (Y.-H.L.); 2 Gachon Institute of Genome Medicine and Science, Gachon University Gil Medical Center, Incheon 21565, Republic of Korea

**Keywords:** fascial circulation exercise, muscle strength, executive performance, functional near-infrared spectroscopy imaging system, prefrontal cortex

## Abstract

**Background**: Evidence linking fascia-oriented rhythmic movement to executive function and prefrontal hemodynamics in older adults remains limited. This pilot study examined the feasibility and preliminary within-subject associations of a four-week Fascial Circulation Exercise (FCE) program in older Korean women. **Methods**: Twelve cognitively screened women (74.3 ± 6.7 years) completed supervised FCE for four weeks. Pre–post assessments included body composition, grip strength, isokinetic knee performance, executive tasks (TMT-A/B, CDT), and task-evoked prefrontal activation measured via functional near-infrared spectroscopy (ΔHbO). Paired *t*-tests with effect sizes were reported. **Results**: Fat mass decreased (−0.71 kg, *p* = 0.016; dz = −0.74), whereas body weight and BMI were unchanged. Selective improvements were observed in knee flexor peak torque and extensor endurance (*p* < 0.05), with no change in grip strength. ΔHbO increased in the orbitofrontal, ventrolateral, and frontopolar regions during executive tasks. Behavioral performance improved in CDT and showed a trend toward improvement in TMT-B. **Conclusions**: Short-term FCE was feasible and was associated with reduced fat mass, selective neuromuscular gains, and increased task-evoked prefrontal oxygenation. The findings are exploratory and support future randomized controlled trials to determine clinical efficacy.

## 1. Introduction

Older adults experience progressive declines in cognitive and functional abilities, ranging from mild deterioration in specific cognitive domains to severe dementia that characterizes pathological aging. Globally, the number of older adults living with dementia is projected to reach nearly 80 million by 2030 [1]. Given this rapid demographic and epidemiological transition, aging-related cognitive impairment has emerged as a major public health concern, imposing substantial socioeconomic burdens worldwide.

Accumulating evidence indicates that physical exercise supports both cognitive and physical functioning in older adults. Exercise interventions have demonstrated improvements in executive function, inhibitory control, and episodic memory, alongside gains in muscle strength, balance, mobility, and endurance [2,3,4,5,6,7]. Furthermore, mobility-related physical activity characteristics have been positively associated with cognitive functioning in cohort research [8], suggesting that habitual movement contributes to maintaining both physical independence and neural resilience in later life.

One physiological mechanism frequently discussed in exercise–cognition research involves cerebral blood flow (CBF), which plays a critical role in sustaining neuronal metabolism and functional integrity. Exercise-induced modulation of cerebral perfusion has been associated with enhanced oxygen and nutrient delivery to cortical tissue [9], and sustained vascular adaptations have been linked to structural preservation and reduced age-related cognitive decline [10,11]. However, the majority of mechanistic evidence has been derived from aerobic and resistance-based exercise paradigms, whereas alternative movement frameworks remain comparatively less investigated in relation to neurofunctional outcomes.

Importantly, movement-induced neurophysiological responses may extend beyond cardiovascular load alone. Beyond systemic circulatory demand, the mechanical and sensory characteristics of movement—such as elasticity, loading variability, multi-directional strain, and sensorimotor stimulation—may contribute to afferent feedback, autonomic modulation, and systemic circulatory dynamics. Thus, examining how distinct movement modalities differentially engage peripheral connective tissues and sensory pathways is essential when considering potential neurofunctional responses beyond conventional exercise models. Within musculoskeletal science, connective tissue extensibility is recognized as a key determinant of the range of motion and movement efficiency of joints [12,13]. Flexibility-oriented interventions may enhance mechanical performance through improved utilization of elastic strain energy [14]. Myofascial release therapy (MRT) is a manual intervention designed to alleviate fascial restrictions and improve soft-tissue mobility [15], and self-myofascial release techniques (SMRTs) extend these principles into active, self-administered formats [16]. Recent evidence syntheses indicate that SMRTs are associated with acute improvements in flexibility and range of motion [17,18]. Short-term changes in peripheral circulation have also been reported following MRT- or SMRT-based interventions [19,20], whereas findings for strength and functional performance outcomes remain heterogeneous [17,18].

These studies have primarily focused on musculoskeletal pain, mobility, and local physiological responses, and relatively few have directly examined higher-order cognitive outcomes [21]. Direct empirical data linking fascia-oriented movement interventions to executive cognitive enhancement or prefrontal activation therefore remain limited. Although direct neurocognitive evidence is scarce, fascia-oriented movement may influence neural function through indirect or candidate pathways, including enhanced proprioceptive afferent input, rhythmically varied elastic loading, breathing-mediated autonomic modulation, and peripheral circulatory changes. However, these mechanisms remain hypothetical and should not be interpreted as established pathways without controlled experimental verification.

Within contemporary fascial fitness theory [22], fascia-oriented movement has been conceptually differentiated from conventional muscle-strengthening paradigms. Whereas resistance training primarily induces contractile adaptations in muscle fibers, connective tissues may require elastic recoil, multi-directional tensile loading, and rhythmically varied mechanical strain to maintain regenerative capacity and elastic responsiveness. Isolated muscle contractions may generate limited deformation within fascial networks; effective fascial stimulation has been proposed to involve rebound dynamics, spring-like loading patterns, and coordinated whole-body kinetic continuity that facilitate mechanotransductive signaling within connective tissue structures [23].

Grounded in these theoretical principles, the present study introduces Fascial Circulation Exercise (FCE) as a structured fascia-oriented movement model. FCE was developed as a rhythmic, multi-planar, low-to-moderate-intensity movement protocol designed to stimulate elastic fascial structures within an active exercise format suitable for older adults. Unlike therapist-assisted MRT, FCE integrates fascial stimulation into standardized, self-performed movement sequences and incorporates coordinated breathing patterns and proprioceptive cueing to support sensorimotor integration. To our knowledge, FCE has not yet been systematically evaluated with respect to neurocognitive outcomes.

In addition to its mechanical components, the current protocol incorporated neurofunctional assessment through executive-task-related performance and monitoring of prefrontal cortical activation. Thus, FCE in this investigation represents a fascia–neurofunctional movement model integrating elastic connective tissue stimulation with concurrent evaluation of executive processing.

Despite increasing interest in integrative movement approaches, empirical evidence remains limited regarding whether short-term fascia-oriented rhythmic exercise is accompanied by measurable changes in executive performance and prefrontal hemodynamics. Accordingly, this single-arm pilot study was designed to evaluate the feasibility and preliminary neurofunctional response patterns associated with a short-term FCE intervention in older women. Using functional near-infrared spectroscopy (fNIRS), we examined task-evoked prefrontal oxygenation during executive processing to characterize initial hemodynamic patterns rather than to establish definitive efficacy. The present findings are intended to provide exploratory evidence regarding the neurofunctional correlates of a fascia-centered movement intervention in aging populations.

## 2. Materials and Methods

### 2.1. Study Design

This study was conducted as a single-arm pilot intervention designed to evaluate the feasibility and preliminary within-subject effects of a short-term Fascial Circulation Exercise (FCE) program on neuromuscular performance, executive function, and task-evoked prefrontal cortical activation in older women. The pilot design was selected to generate effect size estimates and feasibility data for the future development of adequately powered randomized controlled trials (RCTs). Given the exploratory nature of this pilot investigation, no formal a priori sample size calculation was performed. The sample size was determined based on feasibility considerations and prior pilot studies employing functional near-infrared spectroscopy (fNIRS)based neurofunctional assessment in older adults. The primary objective was to estimate preliminary effect sizes and variance parameters to inform future RCT power calculations rather than to establish definitive efficacy.

**Figure 1:**
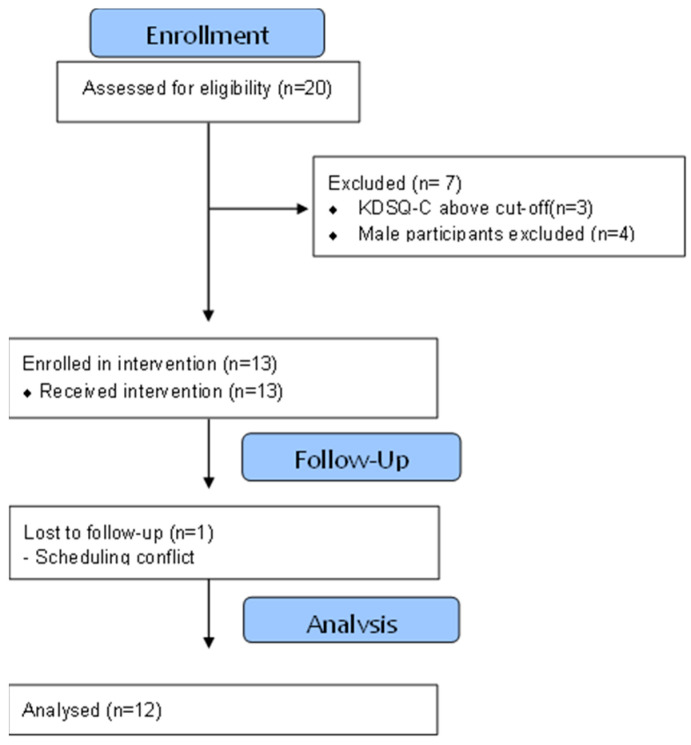
Participant flow diagram of recruitment, screening, intervention, and analysis.

Because this was a single-arm pre–post design without a control group, the study was not intended to determine causal effects of the FCE intervention. Observed changes should therefore be interpreted as preliminary within-subject associations rather than definitive evidence of intervention efficacy. Potential influences such as practice effects, regression to the mean, or nonspecific physical activity effects cannot be excluded. Due to the behavioral and supervised nature of the intervention, participants and instructors were not blinded to the exercise condition. However, cognitive task scoring followed standardized scoring criteria, and fNIRS data were processed using pre-defined analytical pipelines to minimize analytic bias. Statistical analyses were conducted using coded datasets without manipulation of outcome directionality. Accordingly, all findings derived from this pilot investigation should be regarded as hypothesis-generating and exploratory, intended to guide the design and statistical planning of future controlled trials. The participant recruitment and analysis flow are illustrated in Figure 1.

### 2.2. Participants

Participants were recruited from the local community through advertisements and voluntary participation as part of a broader intervention study. Initially, 20 older adults volunteered to participate. Cognitive screening was conducted using the Korean Dementia Screening Questionnaire-Cognition (KDSQ-C; score 3.66 ± 2.18). Three individuals scored above the cut-off value of 6 (score = 7) and were excluded due to potential cognitive impairment. Additional exclusion criteria included a history of psychiatric, neurological, or chronic illnesses. To ensure sample homogeneity and sex consistency, only older women meeting the inclusion criteria were included in the final analysis. Consequently, 12 healthy older Korean women were included in this study (age: 74.3 ± 6.74 years; height: 156.16 ± 7.26 cm; weight: 58.4 ± 9.22 kg; all right-handed). This study was approved by the Ethics Committee of Gachon University Medical Campus (Approval No. #1044396-202503-HR-044-01).

### 2.3. Experimental Settings

Prior to the exercise intervention, the body weight, body fat percentage, skeletal muscle mass, height, and body mass index (BMI) of the participants were evaluated using a body composition analyzer (Inbody 970S; Inbody Co., Ltd., Seoul, Republic of Korea). Additionally, grip strength and knee extensor and flexor muscle performance capacity were measured using a dynamometer (InBody Co., Ltd., Seoul, Republic of Korea) and an isokinetic machine (Humac Norm, CSMi, Stoughton, MA, USA), respectively. To assess cognitive executive function and prefrontal cortex activity response, the participants performed the app-based trail making test black and white and the clock drawing test (JEIOS, Inc., Busan, Korea) equipped with a functional near-infrared spectroscopy (fNIRS) imaging system (NIRSIT, OBELAB, Seoul, Republic of Korea). Two days after the pre-test, the participants were subjected to fascial exercise for 4 weeks under the guidance of a professional instructor. Parameters equal to the pre-test were assessed two days after the completion of the exercise intervention session.

### 2.4. Assessment of Isokinetic Muscle Performance

Isokinetic evaluation of the extensor and flexor muscles was performed using a Cybex device (Humac Norm, Stoughton, MA, USA). Following a general warm-up and a thorough explanation of the test, the participants were seated with their hips locked at an angle of 87° flexion. The participants’ thighs and trunks were securely fastened against the seats with Velcro straps on the thigh and stabilization belts on the trunk. The axes of the dynamometer and the knee joint were aligned. The lower limb was fastened to the dynamometer lever arm at 65% of the leg length, measured from the lateral femoral condyle to the lateral malleolus. A preload corresponding to the weight of the measured limb was applied, and a gravitational adjustment was performed to account for its influence on the torque measurement. During concentric and eccentric motions, the flexor and extensor knee muscles were assessed at velocities of 90°/s and 240°/s, respectively. Prior to measurement, the participants performed two to three repetitions of the exercise to acclimate to the equipment. The dominant limb was measured first, followed by the non-dominant limb after a five-minute rest period. The maximal force curve was accepted, and the knee motion ranged from 5° to 75° (with 0° indicating full extension).

### 2.5. Cognitive Executive Tests Measurement

#### 2.5.1. Trail Making Test Black and White (TMT-B&W)

The two subsets that make up the TMT-B&W are part A and part B (TMT-B&W-A and TMT-B&W-B, respectively). Twenty-five numerals (1–25) are circled in portion A, with even numbers enclosed in a black circle and odd numbers in a white circle. All numbers (2–25) are shown twice in part B, except for 1, which is shown only once in a white circle surrounded by a black and white circle. The TMTB&W and TMT traces were identical. In TMT-B&W-A, the participants were instructed to draw a line to connect the circles in ascending order. TMT B&W-B consists of double the stimuli compared with the TMT B&W-A, with two sets of the 25 numbers in each color (black and white). The participant must connect the numbers in TMT-B&W-B in sequential order while switching between the two color sets. The maximum amount of time permitted was five minutes. Before the test trial, each individual received a practice trial session to ensure that they completely understood how to perform the task. The task completion time was expressed in seconds.

#### 2.5.2. Clock Drawing Test (CDT)

The test was conducted using the following instructions: 1. Provide participants with a pre-drawn circle. 2. Ask the participants to “Please draw the numbers of the clock.” Allow them time to complete it. 3. “Please set the time at ten minutes after eleven.” Instruct the patient to depict a clock face with all numerals and hands and subsequently articulate the indicated time. The images were evaluated according to the Shulman scoring system, which categorizes clock drawings into six groups based on the nature of the abnormalities and the legibility of the design: 5 = normal, 4 = minor visuo-spatial deficits, 3 = incorrect representation of the correct time, 2 = moderate visuo-spatial deficits, 1 = severe visuo-spatial deficits, and 0 = no reasonable depiction of a clock [24].

### 2.6. Task-Evoked fNIRS Signals and Prefrontal Activity Response

All participants were informed about the procedure and operation of the fNIRS system before providing their signed agreement. Brain activity was measured using a portable, multi-channel continuous fNIRS imaging system (NIRSIT, two wavelengths: 780 and 850 nm) equipped with 32 photodetectors and 24 laser sources [25]. A 48-channel probe setup was positioned on the prefrontal cortex, facilitating the mapping of each channel to the corresponding Brodmann regions, including the dorsolateral, ventrolateral, frontopolar, and orbitofrontal cortices. The NIRSIT device was approved as a medical device by the Korea Food and Drug Administration in 2017. To reduce motion artifacts and ambient light noise, the detected signals were processed using a low-pass filter (discrete cosine transform (DCT) 0.05 Hz) and a high-pass filter (DCT 0.005 Hz) [26]. Prior to additional examination, low-quality channels with a signal-to-noise ratio of 30 dB were eliminated. Using the modified Beer–Lambert law (MBLL), the hemodynamic changes for each of the 48 channels throughout each activity were computed [27]. Each task was used to average the individual outcomes, and grand averaging was used to obtain the representative result for each group, as shown by the mean total oxygenated hemoglobin (HbO μm) [28]. Topographical maps were used to depict the activation of different regions of the prefrontal cortex during task performance to illustrate the variations in prefrontal cortex activity. Higher oxygenated hemoglobin levels were indicated by the intensity of the red color, which was also linked to higher activation levels. Conversely, lower oxygenated hemoglobin levels and hypoactivation of the region were indicated by a dark blue tint. To explore the hemodynamic alterations in the prefrontal cortex, the representative value (ΔHbO μm) was extracted by averaging the block-averaged HbO μm in each channel based on the frontal regions. Although the Montréal Neurological Institute (MNI) coordinates varied, regional grouping reliably computed regional representative values for the left and right frontal areas. The normality of ΔHbO μm was tested before and after the intervention using the Shapiro–Wilk and Kolmogorov–Smirnov tests. HbO μm changes during cognitive executive tasks were calculated before and after the fascial exercise intervention using a paired *t*-test. Changes in oxygenated hemoglobin concentration (ΔHbO) were interpreted as indicators of task-evoked hemodynamic modulation within the prefrontal cortex. ΔHbO alterations were not assumed to directly represent clinical cognitive improvement but rather neurophysiological correlates associated with task performance.

### 2.7. Exercise-Based Self-Myofascial Release Intervention

The Fascial Circulation Exercise (FCE) intervention was delivered over four weeks. Participants were scheduled to attend three sessions per week and were required to participate in at least two supervised sessions weekly to ensure adherence. Each session lasted approximately 40–50 min, consistent with physical activity recommendations for older adults. All sessions were conducted under the supervision of a certified movement instructor with experience in fascia-based movement training. Exercise intensity was maintained at a low-to-moderate level and individually adjusted when necessary. Each session consisted of three phases: warm-up (10 min), fascial circulation movement training (25–30 min), and cool-down (5–10 min).

**Figure 2:**
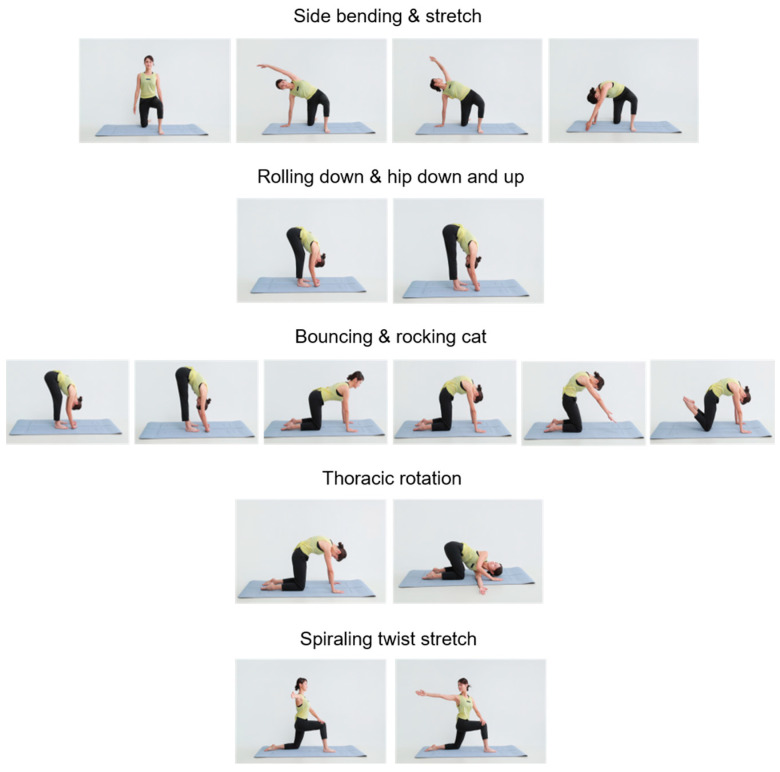
Composition and flow movements of the Fascial Circulation Exercise (FCE). Note: Photographs showing the composition of the Fascial Circulation Exercise (FCE). The FCE consisted of five rhythmic motions collectively termed the “Triple Flow Move”: side bending and stretching; rolling down and hip down-and-up; and cat–camel and transversal rotation (bouncing and rocking cat, thoracic rotation, and spiraling twist stretch).

The FCE was developed by our group, emphasizing neuromyofascial activation via rhythmic, flow-based movement patterns designed to enhance circulation, balance, and proprioceptive awareness. The FCE consisted of five movements collectively termed the “Triple Flow Move” (Figure 2): side bending and stretching; rolling down and hip down-and-up; and cat–camel and transversal rotation (bouncing and rocking cat, thoracic rotation, and spiraling twist stretch).

Each movement has specific functional purposes. Side bending and stretching facilitate intercostal muscle relaxation, spinal alignment restoration, lymphatic flow, and emotional stabilization. Rolling down and hip down-and-up target the posterior fascial line (erector spinae, hamstrings, and calves), supporting posture correction, tension relief, and deep circulation stimulation. Cat–camel and transversal rotation promote thoracic mobility, core stabilization, restoration of back-region sensation, emotional release, and digestive and respiratory function.

The five exercise movements were performed repeatedly across sessions over the four-week intervention period. A detailed session protocol is provided in Appendix A.

### 2.8. Statistical Analysis

Statistical analyses were performed using SPSS for Windows version 29.0 (IBM Corp., Armonk, NY, USA). Pre- and post-intervention differences were analyzed using paired *t*-tests after verification of normality (Shapiro–Wilk test). All values are presented as mean ± standard deviation (SD). Statistical significance was set at *p* < 0.05.

In addition to *p*-values, effect sizes (Cohen’s d for paired comparisons) were calculated to quantify the magnitude of change. Ninety-five percent confidence intervals (95% CI) were reported for mean differences to provide precision estimates. Given the pilot nature of the study, interpretation emphasized effect size estimation and confidence intervals rather than statistical significance alone. No correction for multiple comparisons was applied due to the exploratory design; therefore, the findings should be interpreted cautiously and considered hypothesis-generating.

## 3. Results

### 3.1. A Four-Week FCE Intervention Significantly Reduced Body Fat in Korean Older Women

**Figure 3:**
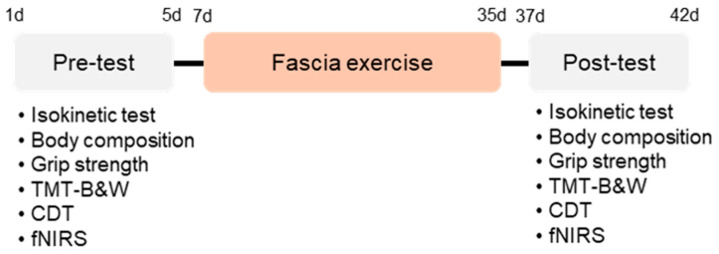
Experimental design and procedure of the four-week FCE intervention. Note: The experimental procedure is illustrated. Before the exercise intervention, participants’ body composition, grip strength, isokinetic knee strength, executive performance, and prefrontal cortex activity were measured. Two days after the pre-test, participants underwent the Fascial Circulation Exercise (FCE) program for four weeks under the supervision of a professional instructor. The same parameters as those assessed in the pre-test were re-evaluated two days after completing the intervention.

**Figure 4:**
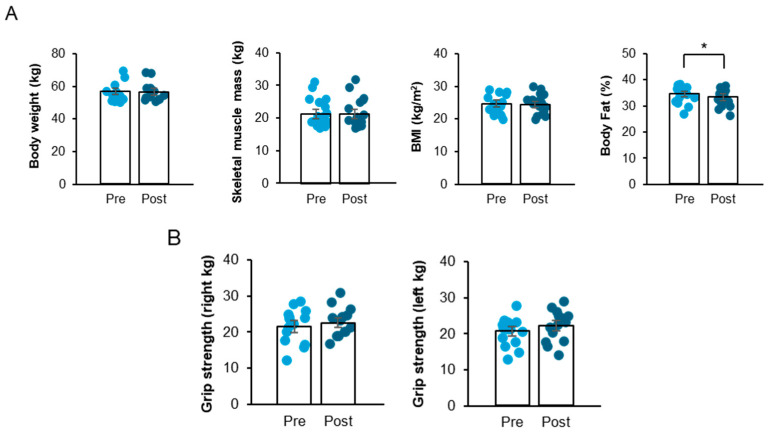
(**A**,**B**). Four-week FCE administration suppressed body fat gain in Korea Older Women. Note: (**A**): Graph showing the body weight, skeletal muscle mass, BMI, and % body fat between before and after the FCE intervention. (**B**): Graph showing the grip strength between before and after the FCE intervention. Data are presented as mean ± SD. * *p* < 0.05 compared with Pre.

To explore the impact of a short-term, 4-week FCE on executive performance and prefrontal cortex responses in Korean older women, body composition, muscle performance, executive tasks, and brain activities were evaluated before and after the intervention (Figure 3). The percentage of body fat significantly decreased following the intervention (Figure 4A; t11 = 2.95, *p* < 0.05).

The corresponding within-subject effect size was calculated (Cohen’s dz), and 95% confidence intervals (CIs) for the mean difference are presented in Table 1. Specifically, fat mass showed a significant reduction (mean difference = −0.71 kg, 95% CI [−1.26, −0.15], *p* = 0.016), with a moderate-to-large effect size (dz = −0.74, 95% CI [−1.32, −0.13]).

Body weight, BMI, and skeletal muscle index did not significantly change after the intervention, and detailed statistics for these variables are presented in Supplementary Table S1. Given the single-arm pilot design (*n* = 12), these findings should be interpreted as preliminary estimates of effect rather than definitive evidence of efficacy.

### 3.2. A Four-Week FCE Intervention Increased Knee Flexor Peak Torque and Knee Extensor Endurance Ratio in Korean Older Women

**Figure 5:**
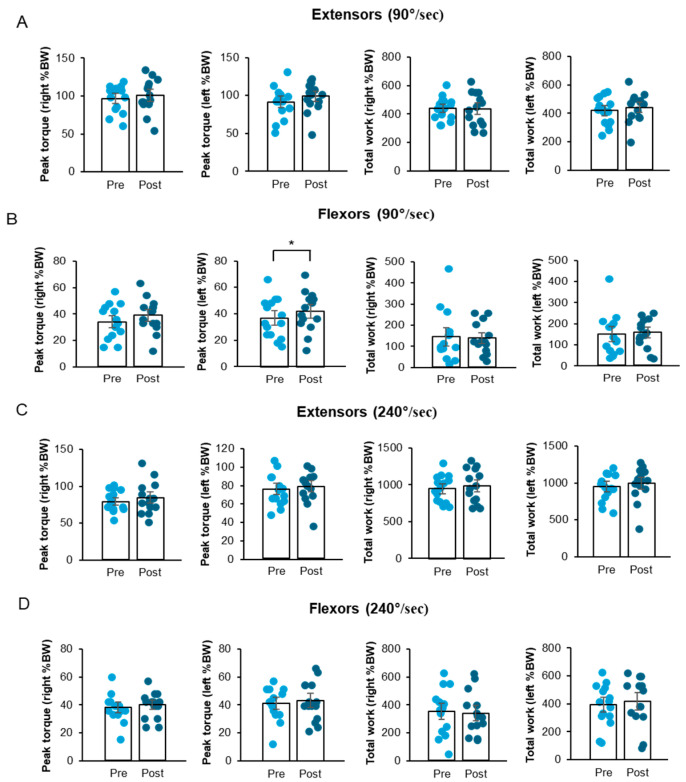

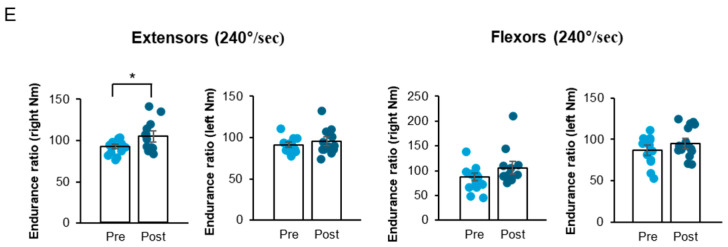
(**A**–**E**). Effects of a four-week FCE intervention on knee flexor peak torque and extensor endurance ratio. Note: (**A**): Graph showing the peak torque and total work of knee extensors at 90°/s before and after the FCE intervention. (**B**): Graph showing the peak torque and total work of knee flexors at 90°/s before and after the FCE intervention. (**C**): Graph showing the peak torque and total work of knee extensors at 240°/s before and after the FCE intervention. (**D**): Graph showing the peak torque and total work of knee flexors at 240°/s before and after the FCE intervention. (**E**): Graph showing the endurance ratio of knee extensors and flexors at 240°/s before and after the FCE intervention. Data are expressed as mean ± SD. * *p* < 0.05 vs. pre-test.

Grip strength and isokinetic knee strength were measured to investigate muscle performance capacity. Right and left grip strengths before and after the FCE intervention were comparable (Figure 4B). In the isokinetic knee strength measurement, the FCE intervention significantly enhanced the peak torque of the left knee flexor muscle at 90°/s (Figure 5B; t_11_ = −2.20, *p* < 0.05) and the endurance ratio of the right knee extensor muscle at 240°/s (Figure 5E; t_11_ = −2.27, *p* < 0.05). Specifically, a statistically significant increase in the peak torque of the left knee flexors was observed at an angular velocity of 90°/s. Furthermore, the endurance ratio of the right knee extensors showed a significant improvement at 240°/s.

Although not all measures reached statistical significance, the intervention consistently showed a trend toward improving various aspects of knee muscular strength (Figure 5A–E). Detailed statistical results for non-significant outcomes are presented in Supplementary Table S1. This finding showed that the FCE intervention, despite its short 4-week duration, resulted in some significant improvements in selected measures of knee extensor and flexor muscle strength in older Korean women, despite the short treatment period.

### 3.3. ΔHbO (Oxygenated Hemoglobin Concentration) Increased in the Ventrolateral Prefrontal Cortex Without Changes in TMT-A Completion Time

**Figure 6:**
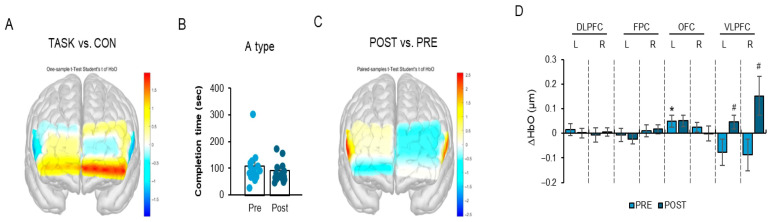

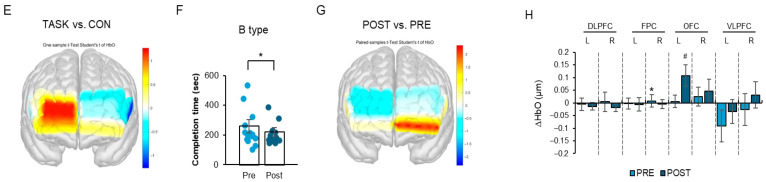
(**A**–**H**). Effects of a four-week FCE intervention on TMT-A and TMT-B performance and changes in ΔHbO in the orbitofrontal (OFC) and ventrolateral prefrontal cortex (VLPFC) of Korean older women. Note: (**A**): Representative heat map of prefrontal cortex activity during TMT-A performance. (**B**): Graph showing TMT-A completion time before and after the FCE intervention. (**C**): Representative heat map of prefrontal cortex activation before and after the FCE intervention during TMT-A performance. (**D**): Graph showing the ΔHbO levels of prefrontal cortex subregions during TMT-A performance before and after the FCE intervention. (**E**): Representative heat map of prefrontal cortex activity during TMT-B performance. (**F**): Graph showing TMT-B completion time before and after the FCE intervention. (**G**): Representative heat map of prefrontal cortex activation before and after the FCE intervention during TMT-B performance. (**H**): Graph showing the ΔHbO levels of prefrontal cortex subregions during TMT-B performance before and after the FCE intervention. Data are expressed as mean ± SD. * *p* < 0.05 vs. during TMT implementation; ^#^ *p* < 0.05 vs. pre-test.

The TMT, parts A and B, was employed as a standardized neuropsychological tool to evaluate executive performance, during which we measured the corresponding prefrontal cortex responses. During the TMT-A task, the left orbitofrontal cortex (OFC) exhibited a significant increase in ΔHbO following the FCE intervention (Figure 6A,D; t_11_ = 2.21, *p* < 0.05). In addition, ΔHbO significantly increased in both the left and right ventrolateral prefrontal cortex (VLPFC) regions (Figure 6C,D; left VLPFC t_11_ = 2.21, *p* < 0.05; right VLPFC t_11_ = 2.57, *p* < 0.05).

However, the completion time of the TMT-A task did not significantly change after the intervention (Figure 6B; t_11_ = 1.12, *p* > 0.05). Non-significant regional activation results are summarized in Supplementary Table S1. Importantly, changes in ΔHbO should not be interpreted as direct evidence of cognitive improvement. Rather, ΔHbO reflects task-evoked hemodynamic responses in prefrontal regions, representing a neurophysiological correlate of cortical engagement. In the absence of concurrent behavioral improvement (i.e., unchanged TMT-A completion time), the observed ΔHbO increase may indicate early neural adaptation or compensatory recruitment rather than measurable enhancement in executive efficiency.

Effect sizes (Cohen’s dz for paired comparisons) and corresponding 95% confidence intervals are reported to contextualize the magnitude and precision of the observed changes, consistent with pilot study reporting standards.

### 3.4. Increased ΔHbO in the Left Orbitofrontal Cortex and Improved TMT-B Performance After FCE

In the TMT-B task, ΔHbO in the right frontopolar cortex (FPC) significantly increased following the intervention (Figure 6E,H; t_11_ = 3.13 *p* < 0.05). In addition, ΔHbO significantly increased in the left orbitofrontal cortex (OFC) (Figure 6E,H; t_11_ = 2.34, *p* < 0.05).

The completion time for the TMT-B task showed improvement after the intervention (Figure 6F; t_11_ = 2.18), approaching statistical significance.

Given the modest sample size (*n* = 12) and the single-arm pilot design, this behavioral trend should be interpreted cautiously. Although increased ΔHbO and improved TMT-B performance were observed concurrently, causality cannot be inferred. The present findings should therefore be considered preliminary patterns intended to inform the design of a future randomized controlled trial (RCT).

All other regional activation results are summarized in Supplementary Table S1.

Collectively, these results suggest that the four-week FCE intervention was associated with increased prefrontal hemodynamic responsiveness during executive task performance; however, the extent to which these neural changes translate into clinically meaningful executive gains remains to be established.

### 3.5. Increased ΔHbO in the Right Ventrolateral Prefrontal Cortex and Improved CDT Performance

**Figure 7:**
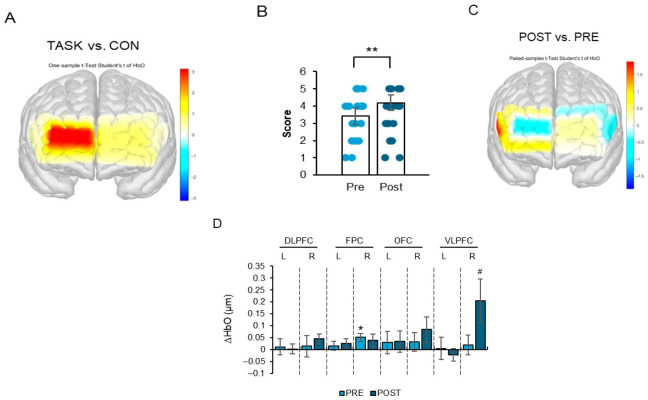
(**A**–**D**). Effects of a four-week FCE intervention on changes in ΔHbO in the right ventrolateral prefrontal cortex (VLPFC) and CDT performance in Korean older women. Note: (**A**): Representative heat map of prefrontal cortex activity during CDT performance. (**B**): Graph showing CDT performance scores before and after the FCE intervention. ** *p* < 0.01 compared with Pre. (**C**): Representative heat map of prefrontal cortex activation before and after the FCE intervention during CDT performance. (**D**): Graph showing ΔHbO levels of prefrontal subregions during CDT performance before and after the FCE intervention. Data are expressed as mean ± SD. * *p* < 0.05 vs. during CDT implementation; ^#^ *p* < 0.05 vs. pre-test.

During CDT task performance, ΔHbO significantly increased in the right frontopolar cortex (FPC) (Figure 7A,D; t_11_ = 2.82, *p* < 0.05). Furthermore, ΔHbO significantly increased in the right ventrolateral prefrontal cortex (VLPFC) after the intervention (Figure 7B–D; t_11_ = 2.18, *p* < 0.05).

CDT performance scores also significantly improved following the FCE intervention (Figure 7B; t_11_ = −3.02, *p* < 0.05).

Non-significant regional activation results are summarized in Supplementary Table S1.

Although the concurrent increase in ΔHbO and improvement in CDT performance suggests a potential functional association, ΔHbO represents a hemodynamic correlate of cortical activation rather than a direct index of cognitive gain. Therefore, the present findings indicate that FCE may be linked to enhanced task-related prefrontal engagement alongside improved visuospatial organization performance; however, mechanistic pathways and long-term clinical relevance require verification in controlled trials.

## 4. Discussion

The present pilot investigation examined the feasibility and preliminary within-subject effects of a four-week Fascial Circulation Exercise (FCE) program on body composition, neuromuscular performance, executive task performance, and task-evoked prefrontal cortical activation in older Korean women. Within the methodological constraints of a single-arm exploratory design, the findings suggest that short-term FCE may be associated with reductions in fat mass, selective improvements in knee muscle performance, and modulation of prefrontal hemodynamic responses during executive task execution.

Given the modest sample size and the number of statistical comparisons conducted in this study, the possibility of Type I error cannot be excluded. Therefore, the present findings should be interpreted cautiously and regarded as preliminary observations rather than definitive evidence of efficacy.

With respect to body composition, fat mass significantly decreased following the intervention, whereas skeletal muscle mass and BMI did not significantly change. This pattern suggests that short-term rhythmic fascia-oriented movement may influence metabolic or compositional parameters without inducing detectable hypertrophic adaptation. Epidemiological and interventional studies indicate that higher levels of physical activity in older adults are associated with improved metabolic regulation and reduced risk of cognitive decline [1,29,30]. However, because the present study lacked a control group and involved a modest sample size, these findings should be interpreted as preliminary associations rather than causal effects.

Selective improvements were observed in left knee flexor peak torque and right knee extensor endurance ratio. These changes may reflect enhanced neuromuscular coordination, altered motor unit recruitment, or improvements in tissue compliance rather than structural muscle hypertrophy. Systematic reviews of self-myofascial release and related fascial techniques report improvements in the range of motion and short-term mechanical function of joints, although effects on strength and performance remain heterogeneous [16,18,21,31,32,33,34,35,36]. Thus, the present findings align with the prior literature suggesting neuromechanical modulation but do not establish definitive strength-enhancing properties of FCE.

A central objective of this study was to explore whether FCE may be associated with modulation of executive performance and corresponding prefrontal cortical activation. During TMT-A, increased ΔHbO was observed in the left orbitofrontal cortex (OFC) and bilateral ventrolateral prefrontal cortex (VLPFC), despite no significant improvement in completion time. During TMT-B, increased ΔHbO in the right frontopolar cortex (FPC) and left OFC was accompanied by a trend toward improved task completion. Similarly, during CDT performance, increased ΔHbO in the right FPC and VLPFC was observed alongside improved CDT scores.

Importantly, increased ΔHbO reflects task-evoked hemodynamic responses measured via fNIRS and represents a neurophysiological correlate of cortical activation rather than direct evidence of functional cognitive enhancement. When behavioral indices do not demonstrate clear improvement, elevated ΔHbO may indicate compensatory neural recruitment or early-stage neurophysiological adaptation rather than improved cognitive efficiency [37]. Therefore, the present findings should not be interpreted as establishing cognitive improvement in a clinical sense but rather as suggesting possible neural modulation associated with task engagement.

The prefrontal regions implicated in this study—including the OFC, VLPFC, and FPC—are known to support working memory, cognitive control, contextual integration, and flexible decision-making processes [37,38,39,40,41,42,43].

The ventrolateral prefrontal cortex (VLPFC), which showed increased task-related ΔHbO in the present study, has been implicated in affect labeling and top–down emotional regulation processes [44,45]. Although the current study did not directly assess emotional regulation or limbic activity, the observed VLPFC modulation may reflect engagement of regulatory control networks during task execution. This interpretation remains speculative and warrants further multimodal investigation.

Prior exercise-based interventions, particularly aerobic and balance-oriented training, have demonstrated modulation of prefrontal activation in older adults [10,46,47]. However, most mechanistic evidence derives from aerobic or resistance paradigms [2,3,10]. Whether fascia-oriented rhythmic movement confers distinct neurophysiological adaptations remains insufficiently investigated.

The distinctive contribution of the present study lies in evaluating a fascia-oriented rhythmic movement paradigm not merely as a musculoskeletal intervention but in parallel with real-time monitoring of prefrontal activation during executive task performance. Previous research on myofascial release and SMRT has primarily focused on the range of motion, pain, and local physiological responses of joints, with limited examination of concurrent neurofunctional responses within task execution contexts. By integrating mechanical characteristics of rhythmic connective tissue loading with task-evoked neuroimaging, this study attempts a conceptual extension toward linking movement modality with cortical engagement patterns. In this sense, FCE should not be interpreted as an established integrative neurofunctional framework; rather, it may represent a potential movement-based approach that engages sensorimotor and cognitive processes, an interpretation that remains exploratory and requires further empirical validation.

Although increased task-related ΔHbO reflects localized cortical oxygenation, movement-induced physiological responses occur systemically. Exercise-related enhancement in cerebral oxygenation is closely linked to global cardiovascular and metabolic regulation [9,10,11]. Therefore, the observed prefrontal activation may reflect both localized neural recruitment and broader systemic circulatory adaptation during rhythmic movement. The present study did not directly assess cerebral blood flow, vascular reactivity, or systemic hemodynamics; consequently, mechanistic interpretations regarding neurovascular coupling remain speculative.

The present study intentionally focused on older women due to accumulating evidence suggesting sex-specific vulnerability to cognitive decline and neuroenergetic alterations following menopause [28,48,49]. However, the absence of male participants limits generalizability. Furthermore, given the modest sample size (*n* = 12), age-stratified analyses were not statistically feasible. Future adequately powered studies should incorporate sex-diverse samples and age subgroup analyses to examine differential responsiveness across aging stages.

It should also be noted that the original study design initially considered a parallel comparison framework involving an FCE-only intervention group and a functional dietary factor group (aged black garlic supplementation). However, due to limitations in sample size and statistical validity at the analysis stage, comparative group analyses were not included in the present manuscript. Consequently, the final report reflects a single-arm pre–post design. This reduction in design scope restricts interpretative breadth, particularly regarding differentiation between exercise-specific effects and potential nutritional interactions. Therefore, the current findings should be understood as preliminary analyses that do not fully reflect the originally intended parallel design. Future studies with adequately powered samples are required to systematically examine independent and interactive effects of movement-based and nutritional interventions.

Several methodological limitations warrant careful consideration. First, the single-arm pilot design without a comparison group prevents causal attribution of observed changes to FCE. Practice effects, regression to the mean, or nonspecific physical activity influences cannot be excluded. Second, the sample size was modest, limiting statistical precision despite reporting effect sizes and confidence intervals. Third, the short intervention duration may not capture longer-term neuromuscular or neuroplastic adaptation. Fourth, ΔHbO provides an indirect index of cortical oxygenation and should not be equated with durable structural or functional brain change.

Taken together, the present findings suggest that short-term FCE may be associated with peripheral neuromuscular modulation and altered prefrontal activation patterns during executive task performance in older women. However, these findings should be regarded as exploratory and hypothesis-generating. Future studies employing adequately powered randomized controlled designs, longer intervention durations, active comparison groups, age-stratified analyses, and multimodal neuromyofascial and neurophysiological assessments are necessary to determine whether fascia-oriented rhythmic movement produces clinically meaningful cognitive or functional benefits beyond those observed with established exercise modalities.

## 5. Conclusions

This pilot study examined the feasibility and preliminary associations of a short-term Fascial Circulation Exercise (FCE) program with body composition, neuromuscular performance, executive task performance, and task-evoked prefrontal cortical activation in older Korean women. A four-week FCE intervention was associated with a reduction in fat mass and selective improvements in knee muscle performance, accompanied by modest changes in executive task measures and increased task-related prefrontal oxygenation assessed using fNIRS.

Importantly, the observed increase in ΔHbO represents a hemodynamic correlate of cortical activation rather than direct evidence of functional cognitive enhancement. When behavioral improvements are limited or modest, altered prefrontal oxygenation may reflect early-stage neurophysiological adaptation or compensatory recruitment rather than established cognitive benefit. Furthermore, exercise-induced modulation of cerebral oxygenation likely occurs within the broader context of systemic cardiovascular and metabolic responses, and localized prefrontal activation should not be interpreted in isolation.

Given the single-arm pilot design, limited sample size, and inclusion of only older women, causal inferences cannot be drawn and generalizability remains restricted. The present findings should therefore be interpreted as exploratory and hypothesis-generating. Nevertheless, the results provide feasibility data and preliminary effect size estimates to inform the design of future randomized controlled trials investigating fascia-oriented rhythmic movement in aging populations.

Future studies incorporating larger and sex-diverse samples, active control conditions, age-stratified analyses, longer intervention durations, and comprehensive neuromyofascial and neurophysiological assessments are required to determine whether FCE produces clinically meaningful functional outcomes. Overall, FCE may represent a movement-based framework warranting further systematic investigation in older adults.

## Figures and Tables

**Table 1:** Changes in body composition after four-week FCE (*n* = 12).

Variable	Pre (Mean ± SD)	Post (Mean ± SD)	Mean Diff (95% CI)	*p*	d(z) (95% CI)
Height (cm)	154.28 ± 5.87	154.50 ± 5.85	0.22 (−0.27, 0.71)	0.347	0.26 (−0.28, 0.79)
Body weight (kg)	56.16 ± 5.58	55.78 ± 5.77	−0.39 (−1.11, 0.34)	0.274	−0.31 (−0.84, 0.24)
Skeletal muscle mass (kg)	19.65 ± 2.41	19.88 ± 2.39	0.23 (−0.03, 0.49)	0.079	0.51 (−0.06, 1.06)
Fat mass (kg)	19.06 ± 2.69	18.36 ± 2.99	−0.71 (−1.26, −0.15)	0.016	−0.74 (−1.32, −0.13)
BMI (kg/m^2^)	23.64 ± 2.27	23.39 ± 2.35	−0.26 (−0.59, 0.08)	0.122	−0.44 (−0.98, 0.12)

## Data Availability

The data supporting the findings of this study are available upon reasonable request from the corresponding author of this article. Owing to ethical and privacy considerations, access to the data may require approval and adherence to institutional guidelines. If applicable, anonymized datasets can be shared for academic and research purposes, subject to compliance with the relevant data protection regulations. Any additional materials, including supplementary documents or codes used in the analysis, can be provided upon request.

## Supplementary Materials

The following supporting information can be downloaded at: https://www.mdpi.com/article/10.3390/life16030458/s1, Table S1: Complete statistical outcomes before and after the FCE intervention.

## Abbreviations

The following abbreviations are used in this manuscript:

ACPT	Acceptance and Commitment Performance Training
BMI	Body Mass Index
CDT	Clock Drawing Test
DCT	Discrete Cosine Transform
DLPFC	Dorsolateral Prefrontal Cortex
FCE	Fascial Circulation Exercise
fNIRS	Functional Near-Infrared Spectroscopy
FPC	Frontopolar Cortex
HbO	Oxygenated Hemoglobin
KDSQ-C	Korea Dementia Screening Questionnaire–C
MBLL	Modified Beer–Lambert Law
MCI	Mild Cognitive Impairment
MNI	Montreal Neurological Institute
MRI	Magnetic Resonance Imaging
MRT	Myofascial Release Technique
OFC	Orbitofrontal Cortex
SMRT	Self-Myofascial Release Technique
SPSS	Statistical Package for the Social Sciences
TMT	Trail Making Test
TMT-B&W	Trail Making Test–Black and White
VLPFC	Ventrolateral Prefrontal Cortex
WHO	World Health Organization

## Appendix A. Fascial Circulation Exercise (FCE) Session Protocol

### Appendix A.1. FCE Intervention Structure

The Fascial Circulation Exercise (FCE) program was delivered over four weeks.

Participants were scheduled to attend three sessions per week and were required to participate in at least two supervised sessions weekly to ensure adherence.

Each session lasted approximately 40–50 min and was conducted under the supervision of a certified movement instructor experienced in fascia-based exercise training.

Exercise intensity was maintained at a low-to-moderate level appropriate for older adults, corresponding to approximately 9–13 on the Borg Rating of Perceived Exertion (RPE) scale.

Movements were adapted individually when necessary to ensure safety and comfort.

### Appendix A.2. Session Composition

**Phase**	**Duration**	**Content**	**Purpose**
Warm-up	10 min	Gentle mobility exercises and breathing coordination	Joint preparation and circulation activation
Fascial circulation training	20–25 min	Triple Flow Move sequence (5 movements)	Fascial elasticity, proprioception, circulation
Cool-down	5–10 min	Stretching and relaxation-based movement	Recovery and parasympathetic activation

### Appendix A.3. Triple Flow Move Components

The FCE program consisted of five rhythmic movements collectively termed the “Triple Flow Move”.

Each movement was performed in a continuous rhythmic sequence for approximately 4–6 min depending on participant tolerance.

**Movement**	**Description**	**Primary Target**
Side bending and stretching	Lateral trunk mobility with rib expansion	Intercostal muscles, spinal alignment
Rolling down and hip down-and-up	Posterior chain release movement	Posterior fascial line circulation
Bouncing and rocking cat	Gentle spinal flexion–extension rhythm	Spinal mobility and neuromuscular coordination
Thoracic rotation	Rotational trunk movement	Thoracic mobility and proprioception
Spiraling twist stretch	Spiral-pattern whole-body movement	Fascial connectivity and balance

### Appendix A.4. Exercise Frequency and Standardization

- Intervention duration: 4 weeks.
- Recommended frequency: 3 sessions/week.
- Required supervised frequency: ≥2 sessions/week.
- Session duration: 40–50 min.
- Intensity: low-to-moderate (RPE 9–13).
- Instructor-guided sessions ensured pacing consistency.

The intervention protocol was designed in accordance with international physical activity recommendations for older adults (WHO, ACSM).
