# Supplementary material for: Short-Term Fascial Circulation Exercise Modulates Task-Related Prefrontal Oxygenation During Executive Tasks in Older Women: An fNIRS Pilot Study
Source: Life (Basel). 2026 Mar 11;16(3):458. doi: 10.3390/life16030458 (PMC13028561; doi:10.3390/life16030458)
Supplement: Supplementary file 1 [file life-16-00458-s001.zip › life-4189002-supplementary.pdf]

Supplementary Table S1. Complete statistical outcomes before and after the FCE intervention.

Domain	Variable	Statistic (t_{11})	Significance
Body composition	Body fat (%)	2.95	$p < 0.05$
Body composition	Body weight	0.49	n.s.
Body composition	BMI	0.15	n.s.
Body composition	Skeletal muscle index	-0.28	n.s.
Grip strength	Right grip strength	0.49	n.s.
Grip strength	Left grip strength	1.46	n.s.
Isokinetic (90°/s flexors)	Left peak torque	-2.20	$p < 0.05$
Isokinetic (90°/s extensors)	Right peak torque	-1.52	n.s.
Isokinetic (90°/s extensors)	Left peak torque	-1.75	n.s.
Isokinetic (90°/s extensors)	Right total work	0.75	n.s.
Isokinetic (90°/s extensors)	Left total work	-1.18	n.s.
Isokinetic (90°/s flexors)	Right peak torque	-1.30	n.s.
Isokinetic (90°/s flexors)	Right total work	1.92	n.s.
Isokinetic (90°/s flexors)	Left total work	1.14	n.s.
Isokinetic (240°/s extensors)	Right peak torque	1.21	n.s.
Isokinetic (240°/s extensors)	Left peak torque	-1.50	n.s.
Isokinetic (240°/s extensors)	Right total work	-0.83	n.s.
Isokinetic (240°/s extensors)	Left total work	-1.15	n.s.
Isokinetic (240°/s flexors)	Right peak torque	0.73	n.s.
Isokinetic (240°/s flexors)	Left peak torque	0.91	n.s.
Isokinetic (240°/s flexors)	Right total work	0.57	n.s.
Isokinetic (240°/s flexors)	Left total work	-0.83	n.s.
Endurance ratio (240°/s extensors)	Right leg	-2.27	$p < 0.05$
Endurance ratio (240°/s extensors)	Left leg	-1.52	n.s.
Endurance ratio (240°/s flexors)	Right leg	-1.46	n.s.
Endurance ratio (240°/s flexors)	Left leg	-1.41	n.s.
Executive performance	TMT-A completion time	1.12	n.s.
